# Steamed and Fermented Ethanolic Extract from* Codonopsis lanceolata* Attenuates Amyloid-*β*-Induced Memory Impairment in Mice

**DOI:** 10.1155/2016/1473801

**Published:** 2016-05-23

**Authors:** Jin Bae Weon, Min Rye Eom, Youn Sik Jung, Eun-Hye Hong, Hyun-Jeong Ko, Hyeon Yong Lee, Dong-Sik Park, Choong Je Ma

**Affiliations:** ^1^Department of Medical Biomaterials Engineering, College of Biomedical Science, Kangwon National University, Chuncheon 200-701, Republic of Korea; ^2^Laboratory of Microbiology and Immunology, College of Pharmacy, Kangwon National University, Chuncheon 200–701, Republic of Korea; ^3^Department of Food Science and Engineering, Seowon University, Cheongju 361-742, Republic of Korea; ^4^Functional Food & Nutrition Division, Department of Agro-Food Resources, Suwon 441-853, Republic of Korea; ^5^Institute of Bioscience and Biotechnology, Kangwon National University, Chuncheon 200-701, Republic of Korea

## Abstract

*Codonopsis lanceolata* (*C. lanceolata*) is a traditional medicinal plant used for the treatment of certain inflammatory diseases such as asthma, tonsillitis, and pharyngitis. We evaluated whether steamed and fermented* C. lanceolata* (SFC) extract improves amyloid-*β*- (A*β*-) induced learning and memory impairment in mice. The Morris water maze and passive avoidance tests were used to evaluate the effect of SFC extract. Moreover, we investigated acetylcholinesterase (AChE) activity and brain-derived neurotrophic factor (BDNF), cyclic AMP response element-binding protein (CREB), and extracellular signal-regulated kinase (ERK) signaling in the hippocampus of mice to determine a possible mechanism for the cognitive-enhancing effect. Saponin compounds in SFC were identified by Ultra Performance Liquid Chromatography-Quadrupole-Time-of-Flight Mass Spectrometry (UPLC-Q-TOF-MS). SFC extract ameliorated amyloid-*β*-induced memory impairment in the Morris water maze and passive avoidance tests. SFC extract inhibited AChE activity and also significantly increased the level of CREB phosphorylation, BDNF expression, and ERK activation in hippocampal tissue of amyloid-*β*-treated mice. Lancemasides A, B, C, D, E, and G and foetidissimoside A compounds present in SFC were determined by UPLC-Q-TOF-MS. These results indicate that SFC extract improves A*β*-induced memory deficits and that AChE inhibition and CREB/BDNF/ERK expression is important for the effect of the SFC extract. In addition, lancemaside A specifically may be responsible for efficacious effect of SFC.

## 1. Introduction

Alzheimer's disease (AD) is the most common progressive neurodegenerative disorder, causing memory and cognition impairment [1]. There are multiple causes of AD and it is likely that some causes have yet to be discovered. The characteristic pathogenesis of AD is accumulation of amyloid-*β*- (A*β*-) containing senile plaques and neurofibrillary tangles in the brain that lead to inflammation in surrounding tissue [2]. The presence of neurofibrillary tangles, composed of hyperphosphorylated tau (a microtubule-associated protein), and senile plaques correlate with cellular dysfunction. A*β* plays a significant role in the development of AD [3–5].

Acetylcholine (ACh) is a neurotransmitter involved in memory and learning processing in the cholinergic system. Acetylcholinesterase (AChE) is the enzyme that decreases ACh levels by hydrolysis. High AChE activity is present in the brains of AD patients [6, 7].

cAMP-response element-binding protein (CREB) also plays an essential role in learning and memory formation. CREB is transcriptionally activated by phosphorylation at Ser-133 [8].

Brain-derived neurotrophic factor (BDNF), a member of the neurotrophin family, has been identified as a target gene of CREB. Downregulation of BDNF expression is associated with memory impairment [9]. CREB and BDNF expressions enhance long-term potentiation in neuronal plasticity [10]. Extracellular signal-regulated kinase (ERK) plays a fundamental role in cell death, cell proliferation, and neuronal plasticity and in activated CREB [11, 12].

*Codonopsis lanceolata *belongs to the Campanulaceae family and has been used as a traditional herbal medicine for the treatment of hypertension and several inflammatory diseases, such as asthma, tonsillitis, and pharyngitis.* C. lanceolata* contains various compounds such as saponins, alkaloids, tannins, steroids, and polysaccharides [13, 14]. Many previous reports have shown that* C*.* lanceolata* has antilipogenic, antiobesity, and anti-inflammatory effects, as well as being able to inhibit the production of TNF-*α* and nitric oxide, the expression of interleukin- (IL-) 3 and IL-6, and LPS-mediated phagocytic uptake in RAW 264.7 cells [15–17].

A cognitive-enhancing effect of steamed and fermented* C. lanceolata *(SFC), against scopolamine-induced memory impairment, has been reported in one of our previous studies [18]. In our present study, we confirm the effect of SFC on A*β*-induced memory deficits in the Morris water maze and passive avoidance tests. Furthermore, the AChE activity and expression of BDNF, CREB, and ERK phosphorylation in the hippocampus of these mice are evaluated.

## 2. Materials and Methods

### 2.1. Plant Materials

The roots of* C. lanceolata* were purchased from Hoengseong Deodeok direct outlet (Heongseong, Gangwon-Do of Korea, lines of longitude: 37° and latitude: 127°) and identified by Dr. Young Bae Seo, a professor of the College of Oriental Medicine, Daejeon University, a voucher specimen (LHY-001E). The* C. lanceolata* was dried in the shade at 20–30°C for 2 days and then steamed using a steam device (Dechang Stainless, Seoul, Korea) 5 times, for 8 hours each, at 90°C. The steamed* C. lanceolata* was aseptically inoculated with approximately 10^6^ CFU/g of* Bifidobacterium longum* (KACC 20587),* Lactobacillus acidophilus* (KACC 12419), and* Leuconostoc mesenteroides* (KACC 12312) (1 : 1 : 1) in distilled water 8 times and subsequently fermented for 48 hours at 30°C.

600 g fermented* C. lanceolata* was extracted in 60 L with 70% (V/V) ethanol (100 g/10 L) for 24 hours during reflux extraction at 80°C. After evaporation, the fermented* C. lanceolata* (yield: 8.78%) was obtained using spray drying.

### 2.2. Chemical Material

Carboxymethyl cellulose (CMC), amyloid-*β*, donepezil, and acetylcholine were supplied by Sigma Aldrich Co. Ltd. (USA).

Primary antibodies (*β*-actin and BDNF) and secondary antibodies (goat-anti-rabbit IgG HRP and goat-anti-mouse IgG HRP) were purchased from Santa Cruz Biotechnology, Inc. (Dallas, USA).

### 2.3. Animals

Ten-week-old ICR mice (males weighing 25–30 g; Dae Han Biolink Co., Eumse-ong, Korea) were maintained in a temperature-controlled room (20 ± 3°C) with a 12/12-hour light-dark cycle and allowed access to commercial pellet feed and water* ad libitum*. Mice were housed 7 per cage (high: 13 cm, *W*: 20 cm, *L*: 25 cm) and allowed to adapt for a 1-week period before the* in vivo* test. All animal experiments in this study were approved by Kangwon National University Institutional Animal Care and Use Committee (KIACUC) (IACUC approval number KW-150706-1) and carried out according to the guidelines for laboratory animals.

### 2.4. A*β* Peptide Injection and Drug Administration

A*β* peptides were dissolved in distilled water and subsequently stored at −20°C. A*β* peptides were incubated at 37°C for 3 days to induce aggregation. Mice were injected in the bregma with a Hamilton microsyringe housing a 26-gauge needle.

SFC (300, 500, and 800 mg/kg) and donepezil (positive control, 1 mg/kg) were dissolved in 0.5% carboxymethylcellulose (CMC) administered orally to mice at a 2 hour before test trial for 4 days with daily for once on the Morris water maze test and the training trial for the passive avoidance test. Sample administration was started 3 days after A*β* peptide injection.

### 2.5. Morris Water Maze Test

The water maze test was performed as previously described with some modifications [19]. The water maze equipment consisted of a circular pool (90 cm in diameter and 40 cm in height), filled to a depth of 30 cm with water and maintained at a temperature of 20 ± 1°C. Areas of the maze were divided into four equal quadrants, and a white escape platform (10 cm in diameter and 26 cm in height) was submerged 1 cm below the surface of the water in the center of one quadrant. The platform was fixed, and starting points were changed on the outside of the pool each day. Mice were allowed an acquisition session in the absence of the platform for 60 seconds. Mice received a 120-second trial session for 4 consecutive days. The escape latency (seconds), the time to locate the platform, and all swimming behaviors of the mice were recorded and analyzed by the Smart (ver. 2.5.21) video-tracking system. After a 24-hour trial session, the platform was removed for the probe trial, and the time spent in the target quadrant was investigated for 60 seconds to determine the memory of the mice.

### 2.6. Passive Avoidance Test

The passive avoidance test was carried out as described in our previous study [18]. The passive avoidance apparatus (Gemini, San Francisco, USA) consisted of two equally sized compartments (17 cm × 12 cm × 10 cm), with an electrifiable grid floor, that were divided by a guillotine door. The mice received two trials, a training trial and test trial. On the first day, mice were allowed a training trial and were initially placed in the light compartment. The door between the two compartments was opened 20 seconds later and an electric foot shock (0.1 mA/10 g body weight, 2 sec duration) was delivered through the grid floor when the mice moved to the dark compartment. The latency time, the time that the mice took to move to the dark compartment, was recorded. After 24 hours, the mice were again placed into the light compartment for the test trial and the latency time was measured up to 180 seconds.

### 2.7. Acetylcholinesterase Activity Determination

Acetylcholinesterase (AChE) activity was measured using the Ellman method with slight modifications [20]. Mice were sacrificed by decapitation. The mouse brain was removed after completion of the behavioral tests, and the hippocampus was dissected from the brain. The hippocampus was rapidly homogenized with sodium phosphate buffer (pH 7.4) and preincubated for 5 minutes at 37°C. The homogenates were stored at −80°C and measured for AChE activity. The reaction mixture contained 33 *μ*L homogenate, 470 *μ*L sodium phosphate buffer, 167 *μ*L DTNB, and 280 *μ*L acetylcholine iodide. Following incubation for 5 minutes, absorbance was measured at 412 nm using a spectrophotometer.

### 2.8. Tissue Preparation and Western Blot Analysis

Mice were sacrificed by decapitation. Mouse brains were promptly collected and the hippocampus was excised 30 minutes after the behavior tests. Mouse hippocampal tissues were homogenized in ice-cold RIPA buffer containing a protease inhibitor cocktail and centrifuged at 13,000 ×g for 20 minutes to remove particulate matter. The supernatants were stored at −80°C and total protein concentrations were measured using the Bradford assay. The supernatants containing 20–50 *μ*g protein were subjected to 15% SDS-PAGE for 2-3 hours at 100 V and transferred to PVDF membrane at 200 V and 60 mA. After transfer, the membrane was blocked in 5% skimmed milk for 1 hour at room temperature and incubated (overnight at 4°C) with primary antibodies: *β*-actin (1 : 2000 dilution), BDNF (1 : 1000 dilution), CREB (1 : 1000 dilution), p-CREB (1 : 500 dilution), ERK (1 : 1000 dilution), and p-ERK (1 : 1000 dilution). Following incubation with primary antibody, the membranes were washed with 0.1% PBST and incubated with the corresponding secondary antibody (goat-anti-rabbit IgG HRP 1 : 2000 dilution for BDNF, donkey-anti-goat IgG HRP 1 : 2000 dilution for p-CREB and p-ERK and goat-anti-mouse IgG HRP 1 : 2000 dilution for CREB, ERK and *β*-actin) for 1 hour at room temperature. Immunoreactive signals were visualized on X-ray with enhanced chemiluminescence (ECL).

### 2.9. UPLC Analysis of Steamed and Fermented* C. lanceolata*

Steamed and fermented* C. lanceolata* was analyzed with a Waters ACQUITY UPLC system (Waters, Milford, MA, USA) that was equipped with the Waters Synapt Mass spectrometry system (Waters, Milford, MA, USA) in order to identify saponin compounds. Analysis was performed on an ACQUITY BEH C_18_ (2.1 × 100 mm, 1.7 *μ*m, Waters, Milford, MA, USA) at 35°C. The mobile phase consisted of water with 0.1% formic acid (67%) and acetonitrile (33%) and was applied at a flow rate of 0.2 mL/min during a run time of 15 mins.

The desolvation gas temperature was set at 100 and 400°C with a desolvation gas flow of 30 and 600 L/h. The capillary voltage was set at 2.5 kV and the cone voltage was up to 45 V.

### 2.10. Statistical Analysis

Statistical analyses were performed using SPSS 1.9. All data from the Morris water maze test, passive avoidance test, and AChE activity values, as well as Western blotting, were performed by one-way ANOVA and Turkey's post hoc test. All experimental data were expressed as the mean ± SEM and *p* < 0.05, *p* < 0.01, and *p* < 0.001 were considered statistically significance.

## 3. Results

### 3.1. The Cognitive-Enhancing Effect of SFC Extract on A*β*-Induced Spatial Memory Impairment

**Figure 1:**
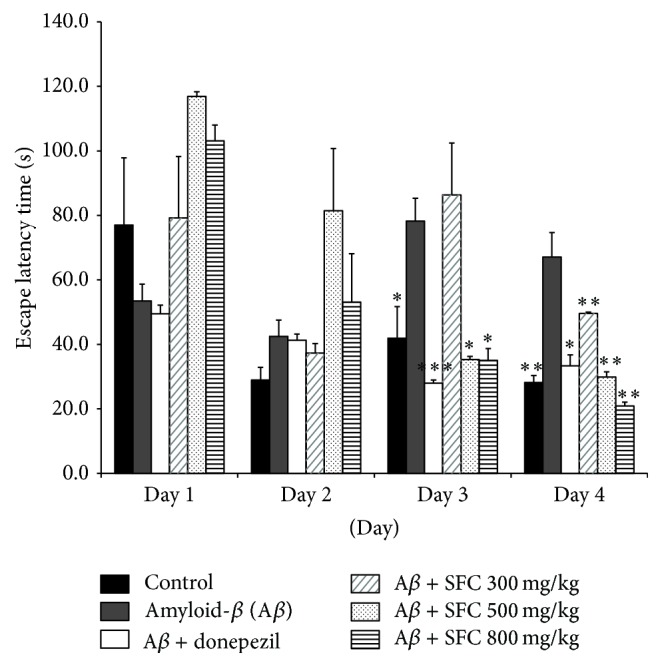
The effect of steamed and fermented* C. lanceolata* on escape latency in A*β*-induced memory impaired mice in the Morris water maze test. The donepezil (1 mg/kg body weight, PO) and the steamed and fermented* C. lanceolata* (SFC) groups (300, 500, and 800 mg/kg body weight, PO) are treated for 90 minutes before cognitive impairment by A*β* administration. The escape latency of each groups' training trial session are presented. The values shown are the mean escape latency ± SEM (*n* = 7). (^*∗*^ *p* < 0.05, ^*∗∗*^ *p* < 0.01, and ^*∗∗∗*^ *p* < 0.001 versus A*β*-treated mice). SFC: steamed and fermented* C. lanceolata*.

**Figure 2:**
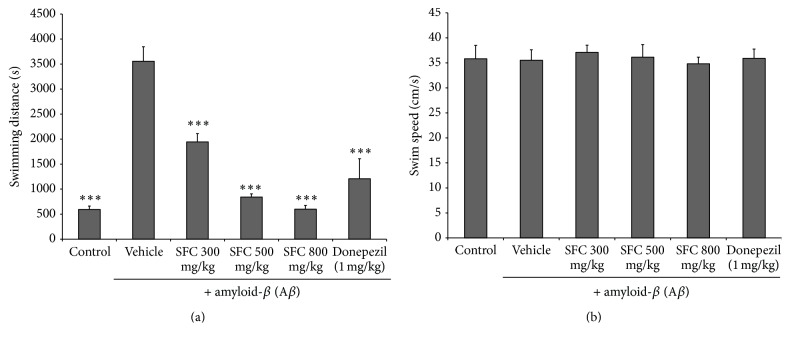
(a) The mean swimming distance to the platform in the Morris water maze test. (b) The mean swimming speed of each group during the 4 trial days in the Morris water maze test. Results are expressed as the mean ± SEM. (*n* = 7) ^*∗∗∗*^ *p* < 0.001 compared with the A*β*-treated group. SFC: steamed and fermented* C. lanceolata*.

**Figure 3:**
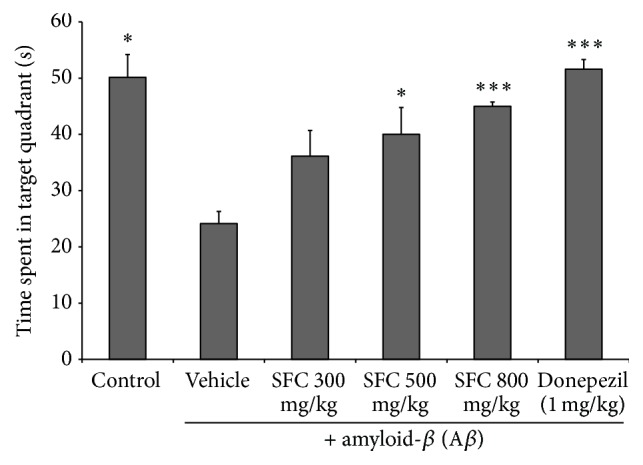
The mean escape latency of each group in the probe trial. The time spent in the target quadrant during the probe trial is presented. Data represent the mean ± SEM. ^*∗*^ *p* < 0.05 and ^*∗∗∗*^ *p* < 0.001 versus the A*β*-treated group. SFC: steamed and fermented* C. lanceolata*.

The effect of SFC extract on A*β*-induced spatial memory impairment was investigated using the Morris water maze test. The control group showed a decrease in escape latency from day 1 to day 4. The escape latency for the untreated memory impaired group significantly increased after day 1. 500 and 800 mg/kg doses of SFC in the treated group showed significantly reduced escape latency during the 2nd and 4th trial days (*p* < 0.005). The donepezil-treated group, a positive control group, showed a decreased escape latency time during the 4 trial days (*p* < 0.005) (Figure 1). In addition, the SFC-treated group showed a decreased mean swimming distance for 4 days compared with the untreated memory impaired group (Figure 2(a)). However, we confirmed that there was no significant difference with the average swimming speed of mice between the groups during the 4-day test (Figure 2(b)). The results suggest that locomotor activity of the mice did not affect escape latency time. In the probe test, the control group showed a significantly increased swimming time in the target quadrant after the platform was removed. The untreated memory impaired group, however, showed a shorter time spent in the target quadrant than the control group, and the swimming time in the target quadrant by the untreated memory impaired mice was significantly increased when treated with SFC (*p* < 0.005) (Figure 3).

**Figure 4:**
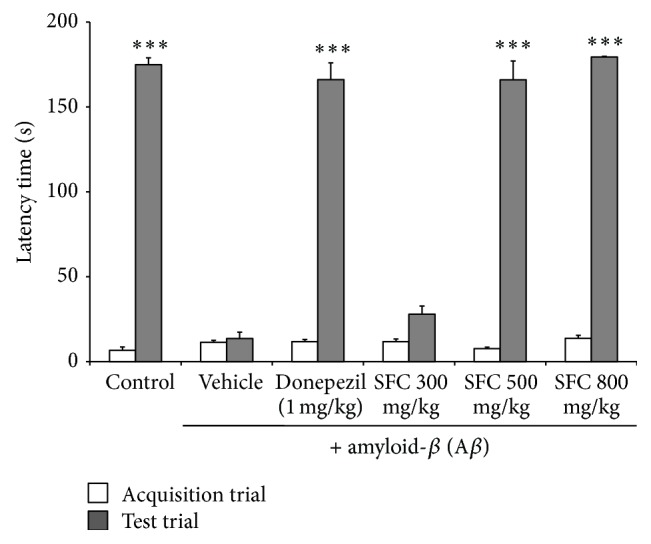
The effect of steamed and fermented* C. lanceolata* on A*β*-induced memory impairment in the passive avoidance test. The latency time to move to the dark compartment was recorded. The mean latency time (s) ± SEM (*n* = 7) ^*∗∗∗*^ *p* < 0.001 compared with the scopolamine group. SFC: steamed and fermented* C. lanceolata*.

We investigated the effect of SFC extract on A*β*-induced memory deficit in the passive avoidance test to assess long-term memory. The untreated memory impaired group showed an increased latency time compared with the control group. The SFC-treated group (500 and 800 mg/kg doses) significantly ameliorated the shortened latency time of the memory impaired mice in a dose-dependent manner (Figure 4). The donepezil-treated group also showed a significant increase in latency time similar to that with the SFC-treated group.

### 3.2. Inhibitory Effect of SFC on Acetylcholinesterase Activity

High AChE activity plays an important role in memory impairment. We analyzed the AChE activity in the hippocampus of A*β*-induced memory impaired mice in order to assess the effect of SFC (Table 1). The AChE activity was significantly increased in the hippocampus of these memory impaired mice as compared with the control group. SFC treatment significantly inhibited AChE activity at the doses of 500 and 800 mg/kg, when compared with the untreated memory impaired mice. Donepezil, used as positive control, also showed a decrease in the AChE activity of memory impaired mice.

### 3.3. The Effect of SFC on BDNF Expression, CREB, and ERK Phosphorylation

The activation of CREB and BDNF improved long-term memory. In addition, the ERK pathway is known to exert memory function. Therefore, in this study we decided to evaluate the effect of SFC on BDNF, p-CREB, and p-ERK expression in the hippocampus using Western blot analysis.

**Figure 5:**
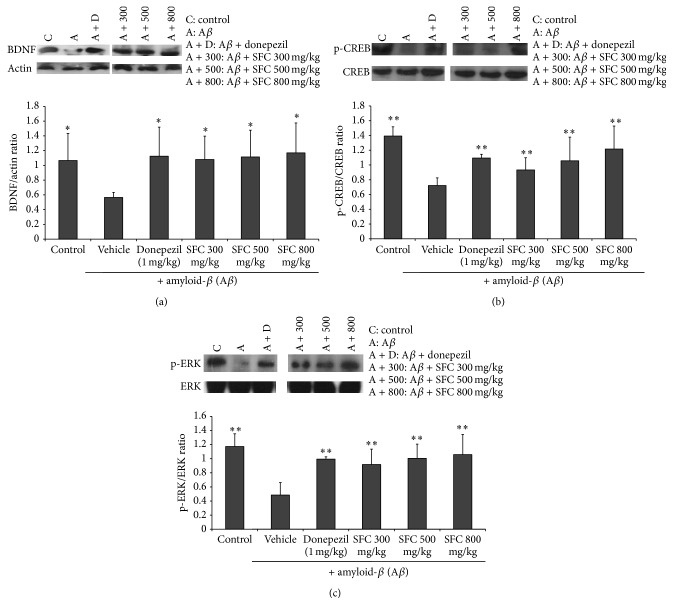
The effect of steamed and fermented* C. lanceolata* on BDNF, CREB, and ERK signaling in the hippocampus by Western blot analysis. Data represent the mean ± SD. ^*∗*^ *p* < 0.05 and ^*∗∗*^ *p* < 0.01 versus the A*β*-treated group. SFC: steamed and fermented* C. lanceolata*.

As shown in Figure 5, the hippocampal expression of BDNF, p-CREB, and p-ERK in the untreated memory impaired mice was lower than that of the control group. SFC (300, 500, and 800 mg/kg, PO) treatment significantly increased expression of all three proteins.

p-ERK expression induces CREB phosphorylation with the increase of BDNF activation in the hippocampus. These results indicate that BDNF activation and CREB and ERK phosphorylation can be suppressed by A*β*-treatment of mice and that the SFC-induced increase in BDNF and p-CREB expression may be dependent on p-ERK signaling.

### 3.4. UPLC-Q-TOF-MS Analysis of Steamed and Fermented* C. lanceolata*

**Figure 6:**
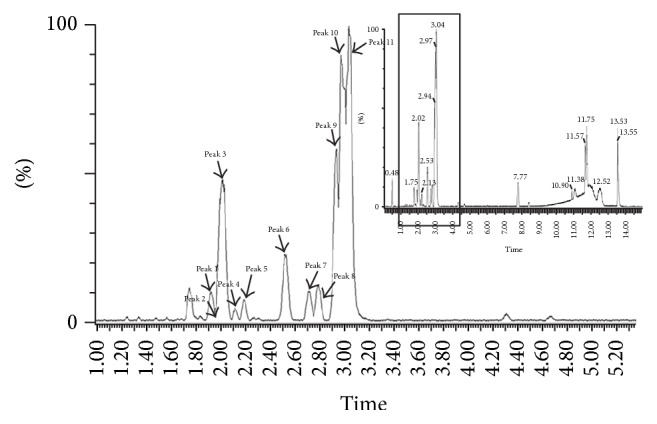
Total ion chromatogram of saponin compounds in steamed and fermented* C. lanceolate*: (1) lancemaside C, (2) lancemaside C, (3) lancemaside B, (4) lancemaside D, (5) unknown, (6) lancemaside E, (7) unknown, (8) unknown, (9) foetidissimoside A, (10) unknown, and (11) lancemaside A.

Saponin compounds are known to be the main compounds within* C. lanceolata*. Therefore, SFC extract was analyzed by UPLLC-Q-TOF to identify the specific saponin compounds present. The total ion chromatogram is shown in Figure 6. Compounds were detected within 5 minutes and were identified according to characteristic* m*/*z* values of mass spectrometry. Retention time and calculated mass data of the compounds is given in Table 2. The results of the UPLC-Q-TOF-MS analysis indicate that lancemasides A, B, C, D, E, and G and foetidissimoside A are the main compounds contained in SFC. We did not identify the compounds from peaks 5, 7, 8, and 10 and further study by NMR was needed to identify these peaks.

## 4. Discussion

Our previous studies confirmed that SFC attenuates memory impairment induced by scopolamine in mice [18]. Scopolamine inhibits cholinergic activity and produces memory impairment. Pathophysiology of AD includes not only cholinergic blockade but also A*β* deposition in the brain. A*β* is a 40–42 amino acid peptide fragment of amyloid precursor protein (APP). Accumulation of A*β* deposits causes the degeneration of cholinergic neuron function and oxidative stress. In this study, we evaluated the effect of SFC on A*β*-induced memory impairment in mice using the Morris water maze and passive avoidance tests. The Morris water maze is the most widely used behavioral test to study hippocampal-dependent spatial learning and memory in mice [19]. The passive avoidance test is also known as a fear-aggravated test to assess long-term memory retention based on the administration of an aversive stimulus such as a foot shock [21].

We confirmed that SFC improves memory and learning in mice with A*β*-induced deficits using the Morris water maze and passive avoidance tests. SFC significantly reduced escape latency time from day 2 to day 4, meaning that long-term memory impairment improved in the Morris water maze test. In the passive avoidance test, SFC also increased the latency time shortened by A*β*-induced memory impaired mice. These results imply that SFC has a cognitive effect on A*β* treatment-induced memory impairment. Result of mean swimming speed was indirectly suggested that* C. lanceolate* extract was not affected on health and locomotor of mice. In this study, we did not observe any adverse health effect after administering the drug.

Previous studies have confirmed that A*β* affects AChE activity. Ach was hydrolyzed by AChE at central cholinergic synapses and A*β* increased AChE activity by the reduction of gene expression of muscarinic M1 receptor in hippocampus [22]. The increased neurotoxicity from the A*β*-AChE complexes induced an increase in intracellular Ca^2+^ and mitochondrial membrane potential loss in hippocampal neurons [23, 24]. In this study, the hippocampus of memory impaired mice treated with SFC was observed to have lower AChE activity in a dose-dependent manner. This result suggests that the effect of SFC may be associated with the muscarinic cholinergic receptor and may reverse cognitive impairment by affecting AChE activity in brain.

The high level of A*β* accumulation interfered with neuronal activity by suppressing BDNF expression and phosphorylation of p-CREB [25]. The activation of CREB and BDNF plays an important role in the learning and memory process. Phosphorylation of CREB in order to switch on its transcriptional activity leads to an upregulation of many specific target genes, including BDNF (phosphorylated at Ser-133) [26]. Upregulation of BDNF gene expression enhances LTP and memory formation at hippocampal and cortical synapses [27, 28].

We investigated the effect of SFC on CREB phosphorylation and BDNF expression in A*β*-injected mice by Western blot analysis. We confirmed that SFC increases BDNF expression and p-CREB levels in hippocampus of these mice.

Signaling of ERK, a member of the mitogen-activated protein kinase (MAPK) superfamily, also has an important role in learning and memory. ERK1/2 signaling mediates the activation of CREB, which participates in the protein kinase A- (PKA-) dependent LTP. Administration of SFC increased ERK signaling in the hippocampus of A*β*-induced memory impaired mice.

In the present study, saponin compounds in SFC were analyzed by UPLC-Q-TOF-MS. Seven compounds present, characterized according to MS data, are lancemasides A, B, C, D, E, and G and foetidissimoside A. Among the seven saponin compounds, lancemaside A is a major saponin that represents the pharmacological activities of* Codonopsis lanceolate* [29]. Lancemaside A ameliorated scopolamine-induced memory and learning deficits in mice on the passive avoidance, Y-maze, and Morris water maze tasks and lanscemaside A inhibited AChE activity and induced BDNF and p-CREB expression in the brain [30].

The results presented herein suggest that the cognitive-enhancing effect of SFC in behavioral tests correlates with the activation of the p-ERK/p-CREB/BDNF pathway, which plays an important role in learning and memory formation. In addition, the effect of SFC may be associated with the action of the compound lancemaside A contained within.

Steamed and fermented* C. lanceolata* improved the amyloid-*β*-induced memory deficit during behavioral tests. In addition, fermented* C. lanceolata* inhibited AChE activity and increased p-CREB and BDNF expression.

## 5. Conclusion

In summary, we investigated the cognitive-enhancing effect of steamed and fermented* C. lanceolata* in the Morris water maze and passive avoidance tests. Steamed and fermented* C. lanceolata* ameliorated amyloid-*β*–induced memory impairment and this effect appears to be mediated via the inhibition of AChE activity and the activation of BDNF, p-CREB, and ERK signaling. Further studies will be conducted in order to determine the role of fermented* C. lanceolata* on the CREB and BDNF expression pathway. Thus, steamed and fermented* C. lanceolata* could be a potential therapeutic agent for the prevention and treatment of neurodegenerative diseases such as AD.

## Figures and Tables

**Table 1:** The inhibition of steamed and fermented *C. lanceolata* on acetylcholinesterase (AChE) activity in the hippocampus of mice. Data represent the mean ± SD. ^*∗*^ *p* < 0.05 versus the A*β*-treated group. SFC: steamed and fermented *C. lanceolata*.

Groups		U/mg protein
Control		1.59 ± 0.17^*∗*^

Scopolamine		2.54 ± 0.76

Donepezil		1.85 ± 0.77^*∗*^

SFC	300 mg/kg	2.29 ± 0.42
	500 mg/kg	1.35 ± 0.25^*∗*^
	800 mg/kg	1.20 ± 0.37^*∗*^

**Table 2:** Characterization of saponin compounds from SFC.

Peak	*t*_*R*_ (min)	*m*/*z* ES(−)	Assignment
1	1.93	1219.5748	Lancemaside C
2	1.99	1205.5591	Lancemaside G
3	2.02	1351.617	Lancemaside B
4	2.12	1087.5325	Lancemaside D
5	2.19	1189.5642	Unknown
6	2.53	1351.617	Lancemaside E
7	2.71	1219.5748	Unknown
8	2.78	1189.5642	Unknown
9	2.94	1057.5219	Foetidissimoside A
10	2.98	1189.5642	Unknown
11	3.02	1189.5642	Lancemaside A

## List of Abbreviations

SFC: Steamed and fermented* C. lanceolata*
AChE: Acetylcholinesterase
BDNF: Brain-derived neurotrophic factor
CREB: Cyclic AMP response element-binding protein
ERK: Extracellular signal-regulated kinase
UPLC-Q-TOF-MS: Ultra Performance Liquid Chromatography-Quadrupole-Time-of-Flight Mass Spectrometry.

## References

[1] Crapper D. R., DeBoni U. (1978). Brain aging and Alzheimer's disease. *Canadian Psychiatric Association Journal*.

[2] Collerton D. (1986). Cholinergic function and intellectual decline in Alzheimer's disease. *Neuroscience*.

[3] Sadigh-Eteghad S., Sabermarouf B., Majdi A., Talebi M., Farhoudi M., Mahmoudi J. (2015). Amyloid-beta: a crucial factor in Alzheimer's disease. *Medical Principles and Practice*.

[4] Esch F. S., Keim P. S., Beattie E. C. (1990). Cleavage of amyloid *β* peptide during constitutive processing of its precursor. *Science*.

[5] Palop J. J., Mucke L. (2010). Amyloid-Β-induced neuronal dysfunction in Alzheimer's disease: from synapses toward neural networks. *Nature Neuroscience*.

[6] Coyle J. T., Price D. L., DeLong M. R. (1983). Alzheimer's disease: a disorder of cortical cholinergic innervation. *Science*.

[7] Ballard C. G. (2002). Advances in the treatment of Alzheimer's disease: benefits of dual cholinesterase inhibition. *European Neurology*.

[8] Saura C. A., Valero J. (2011). The role of CREB signaling in Alzheimer's disease and other cognitive disorders. *Reviews in the Neurosciences*.

[9] Bekinschtein P., Cammarota M., Izquierdo I., Medina J. H. (2008). Reviews: BDNF and memory formation and storage. *The Neuroscientist*.

[10] Calabrese F., Guidotti G., Racagni G., Riva M. A. (2013). Reduced neuroplasticity in aged rats: a role for the neurotrophin brain-derived neurotrophic factor. *Neurobiology of Aging*.

[11] Ma Q.-L., Harris-White M. E., Ubeda O. J. (2007). Evidence of A*β*- and transgene-dependent defects in ERK-CREB signaling in Alzheimer's models. *Journal of Neurochemistry*.

[12] Perry G., Roder H., Nunomura A. (1999). Activation of neuronal extracellular receptor kinase (ERK) in Alzheimer disease links oxidative stress to abnormal phosphorylation. *NeuroReport*.

[13] Yongxu S., Jicheng L. (2008). Structural characterization of a water-soluble polysaccharide from the roots of *Codonopsis pilosula* and its immunity activity. *International Journal of Biological Macromolecules*.

[14] Ushijima M., Komoto N., Sugizono Y. (2008). Triterpene glycosides from the roots of *Codonopsis lanceolata*. *Chemical & Pharmaceutical Bulletin*.

[15] Byeon S. E., Choi W. S., Hong E. K. (2009). Inhibitory effect of saponin fraction from *Codonopsis lanceolata* on immune cell-mediated inflammatory responses. *Archives of Pharmacal Research*.

[16] Ryu H. S. (2009). Effect of Codonopsis lanceolatae extracts on mouse IL-2, IFN-, IL-10 cytokine production by peritoneal macrophage and the ratio of IFN-, IL-10 cytokine. *The Korean Journal of Food And Nutrition*.

[17] Li J. P., Liang Z. M., Yuan Z. (2007). Triterpenoid saponins and anti-inflammatory activity of *Codonopsis lanceolata*. *Die Pharmazie*.

[18] Weon J. B., Yun B.-R., Lee J. (2014). Cognitive-enhancing effect of steamed and fermented *Codonopsis lanceolata*: a behavioral and biochemical study. *Evidence-Based Complementary and Alternative Medicine*.

[19] Morris R. (1984). Developments of a water-maze procedure for studying spatial learning in the rat. *Journal of Neuroscience Methods*.

[20] Ellman G. L., Courtney K. D., Andres V., Feather-Stone R. M. (1961). A new and rapid colorimetric determination of acetylcholinesterase activity. *Biochemistry & Pharmacology*.

[21] O'Keefe J., Nadel L. (1978). *The Hippocampus as a Cognitive Map*.

[22] Jin C.-H., Shin E.-J., Park J.-B. (2009). Fustin flavonoid attenuates *β*-amyloid (1–42)-induced learning impairment. *Journal of Neuroscience Research*.

[23] Melo J. B., Agostinho P., Oliveira C. R. (2003). Involvement of oxidative stress in the enhancement of acetylcholinesterase activity induced by amyloid beta-peptide. *Neuroscience Research*.

[24] Reyes A. E., Chacón M. A., Dinamarca M. C., Cerpa W., Morgan C., Inestrosa N. C. (2004). Acetylcholinesterase-A*β* complexes are more toxic than A*β* fibrils in rat hippocampus: effect on rat *β*-amyloid aggregation, laminin expression, reactive astrocytosis, and neuronal cell loss. *American Journal of Pathology*.

[25] Tong L., Thornton P. L., Balazs R., Cotman C. W. (2001). *β*-amyloid-(1–42) impairs activity-dependent cAMP-response element-binding protein signaling in neurons at concentrations in which cell survival is not compromised. *The Journal of Biological Chemistry*.

[26] Kida S. (2012). A functional role for CREB as a positive regulator of memory formation and LTP. *Experimental Neurobiology*.

[27] Panja D., Bramham C. R. (2014). BDNF mechanisms in late LTP formation: a synthesis and breakdown. *Neuropharmacology*.

[28] Bramham C. R., Messaoudi E. (2005). BDNF function in adult synaptic plasticity: the synaptic consolidation hypothesis. *Progress in Neurobiology*.

[29] Shirota O., Nagamatsu K., Sekita S. (2008). Preparative separation of the saponin lancemaside A from *Codonopsis lanceolata* by centrifugal partition chromatography. *Phytochemical Analysis*.

[30] Jung I.-H., Jang S.-E., Joh E.-H., Chung J., Han M. J., Kim D.-H. (2012). Lancemaside A isolated from *Codonopsis lanceolata* and its metabolite echinocystic acid ameliorate scopolamine-induced memory and learning deficits in mice. *Phytomedicine*.

